# Gene‐level genome‐wide association analysis of suicide attempt, a preliminary study in a psychiatric Mexican population

**DOI:** 10.1002/mgg3.983

**Published:** 2019-10-02

**Authors:** Thelma Beatriz González‐Castro, José Jaime Martínez‐Magaña, Carlos Alfonso Tovilla‐Zárate, Isela Esther Juárez‐Rojop, Emmanuel Sarmiento, Alma Delia Genis‐Mendoza, Humberto Nicolini

**Affiliations:** ^1^ División Académica Multidisciplinaria de Jalpa de Méndez Universidad Juárez Autónoma de Tabasco Mexico City Mexico; ^2^ División Académica Multidisciplinaria de Ciencias de la Salud Universidad Juárez Autónoma de Tabasco Villahermosa Mexico; ^3^ Instituto Nacional de Medicina Genómica (INMEGEN) Secretaria de Salud Mexico City Mexico; ^4^ División Académica Multidisciplinaria de Comalcalco Universidad Juárez Autónoma de Tabasco Comalcalco Mexico; ^5^ Hospital Psiquiátrico Infantil "Dr. Juan N. Navarro" Mexico City Mexico

**Keywords:** genome‐wide, Mexican population, psychiatrics diseases, suicide attempt

## Abstract

**Background:**

Evidence suggests that liability for suicide behavior is heritable; additionally, suicide has been partly related to other psychiatric disorders. Nevertheless, most of the information reported so far address Caucasian and Asian individuals. Hence, our aim was to conduct a gene‐level association study in Mexican psychiatric individuals diagnosed with suicide attempt.

**Methods:**

We recruited 192 individuals from two clinical centers in Mexico. All participants were born in Mexico and had Mexican parents and grandparents. Direct genotyping was performed using the commercial platform Infinium PsychArray BeadChip. A *p*‐value lower than 1e‐05 was considered as gene‐level significant and a *p*‐value lower than 1e‐04 was considered as gene‐level nominal significant.

**Results:**

Our analyses showed that *SCARA5* was associated to suicide intent at a gene‐level with statistical significance (*p*‐value = 1.12e‐6). Other genes were nominally associated with suicide attempt: *GHSR* (*p*‐value = 0.0004), *RGS10* (*p*‐value = 5.13e‐5), and *STK33* (*p*‐value = 3.62e‐5). Regarding gene variant analyses, the SNPs with a statistical association (*p* > .05) were rs561361616, rs1537577, rs11198999 for *RGS10*, and rs11041981, rs11041993, rs11041994, rs11041995, rs11041997, rs10840083, rs10769918 for *STK33*. For these genes, previous studies have associated *SCARA5* with depression, *GHSR* with alcohol dependence and depression, and *RGS10* with schizophrenia and depression. To date, *STK33* has not been associated with any psychiatric disorder.

**Conclusion:**

Our outcomes revealed that *SCARA5*, *GHSR*, *RGS10* and *STK33* could be considered as risk biomarkers for suicide attempt behavior in our Mexican psychiatric sample. We recommend to perform larger scale analyses to have conclusive results.

## Graphical abstract

One hundred and ninety‐two individuals were recruited from two clinical centers in Mexico. Our analyses showed that *SCARA5*, *GHSR*, *RSG10,* and *STK33* were associated to suicide intent at a gene‐level. We recommend to perform larger scale analyses to have conclusive results.

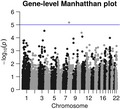

## 1 INTRODUCTION

Suicide is an important health problem all over the word; nearly 800,000 suicide deaths were observed in 2016 worldwide (Organiztion, 2019). It has been reported that more than 80% of individuals who died by suicide had at least one psychiatric diagnosis (Galfalvy et al., 2015, 2013). For instance, it has been suggested that individuals with schizophrenia and bipolar disorder have higher rates of suicide (Mirkovic et al., 2016; Zai et al., 2015) mainly due to processes of internalizing (as posttraumatic stress, depression) and externalizing (as substance use, antisocial personality) problems (Schneider & Grozinger, 2010).

It has also been observed that suicide has a prominent genetic component (Sokolowski, Wasserman, & Wasserman, 2016a; Stein et al., 2017; Willour et al., 2012). For example, Fu et al., addressed the relation between suicidal ideation (SI) and suicidal behavior (SB) in 3,372 male twin pairs from the Vietnam Era Twin Registry and they found that monozygotic (MZ) twins were more likely to be concordant for suicide attempts than dizygotic (DZ) twins; even after adjusting for other risk factors of SB, psychiatric diagnoses were not discarded for the inclusion criteria. They reported heritability of 43% for SI and 30% for suicide attempt (SA; Fu et al., 2002). Another example is the study by Glowinski et al. that reported heritability of 38% in a representative sample of 3,416 female suicide‐attempter twins from the Child Semi‐Structured Assessment for the Genetics of Alcoholism, which includes a detailed SB section. In addition, they observed a concordance rate of 25% in MZ versus 12.8% in DZ twins. Nevertheless, the precise genetic predisposition in these studies has not been fully understood yet (Glowinski et al., 2001). Therefore, identifying genetic components that contribute to develop suicide behavior/attempt in individuals with psychiatric disorders will provide an insight into the biology underlying these diseases (Sokolowski et al., 2016a; Sokolowski, Wasserman, & Wasserman, 2016b).

Research in genetics has focused on searching for candidate genes that could lead to a better understanding of psychiatric diseases and could work as possible biomarkers that help prevent, diagnose, or treat these diseases (Eftekharian et al., 2018; Genis‐Mendoza et al., 2017; Lopez‐Narvaez et al., 2015; Molina‐Guzman et al., 2017). Nevertheless, association studies of candidate genes frequently generate contradictory results; for this reason, technical improvements have led to genome‐wide association studies (GWAS) in order to locate genes or loci using the whole genome of individuals (Kimbrel et al., 2018; Lasky‐Su & Lange, 2010; Melroy‐Greif, Gizer, Wilhelmsen, & Ehlers, 2017). Willour et al. (2012) conducted a GWAS in a sample of 2,698 individuals with bipolar disorder, of whom 1,201 subjects had previous suicide attempts; they found an association with marker rs300774 located in chromosomal region 2p25 (Willour et al., 2012). Moreover, Perlis et al., observed the strongest association in a suicide attempt group with bipolar disorder that came from the intergenic chromosome 10 marker rs1466846 (Perlis et al., 2010). However, a small study used GWAS to identify candidate genes and did not find any variants that reached the traditional threshold for significance, but the expression array identified a cluster of genes involved in neuroimmune function (Galfalvy et al., 2013). GWAS have been an excellent tool to find signals related to genetic factors affecting diseases; nevertheless, these genetic signals are made through individualized variants; however, GWAS do not measure the possible effects of one particular gene, which is a limitation (Powers et al., 2016; Wang, Jia, Wolfinger, Chen, & Zhao, 2011). Despite the important information that has been obtained through GWAS, the outcomes mainly reflect Caucasian or Asian populations, as there are not enough data published for Latin American populations (Melroy‐Greif et al., 2017; Mullins et al., 2014; Sokolowski, Wasserman, & Wasserman, 2014).

Contrary to the rest of the word, in Mexico, suicide rates have increased over the past 40 years (Borges et al., 2019). Therefore, several genetic association studies have been performed recently in this population (Genis‐Mendoza et al., 2017; Molina‐Guzman et al., 2017); however, no GWAS studies have been performed in Mexican population. Hence, we performed a gene‐level genome‐wide association analysis in Mexican individuals with a psychiatric diagnosis and SA. Additionally, we conducted a prediction and functional analysis of the associated variants.

## 2 MATERIALS AND METHODS

### 2.1 Ethical compliance

The protocol was approved by the ethics committee of research of the “Instituto Nacional de Medicina Genómica” (approbation number: 23/2015/I). All individuals included in the study signed an informed consent and the protocols were developed in accordance with the Helsinki Declaration.

### 2.2 Sample

Participants were recruited from two clinical centers: Regional Hospital Dr. Desiderio G. Carbajal in Tabasco State (*n* = 16), and from the Carracci Medical group in Mexico City (*n* = 176). The total sample consisted of 192 individuals of 18 to 55 years of age, 100 were males (52.08%) and 92 were females (49.92%). All individuals included were born in Mexico and had Mexican descendance of at least two generations.

### 2.3 Cases

The case group consisted of individuals who attended the service of psychiatry in any of the hospitals previously mentioned and were diagnosed with at least one SA. Initially, clinical interviews helped us to determine the most relevant symptoms, diagnoses were determined using the DSM‐IV criteria (Diagnostic and Statistical Manual of Mental Disorder Fourth Edition) in which disorders pertaining to Axis I were considered; those diagnoses were confirmed by at least two experienced psychiatrists through the Spanish version of DIGS (Diagnostic Interview for Genetic Studies). As exclusion criteria, we considered major depressive episodes or anxiety condition, a lifetime history of severe head trauma or central nervous system disorder and mental retardation, and individuals under 18 years of age. First, we gathered 125 candidates, but only 37 patients met the inclusion criteria of SA, for more detail see Figure S1. In these 37 suicide attempters, the lifetime DSM‐IV diagnoses were paranoid schizophrenia 72.97% (range age: 18–38; male/female: 6/4) and bipolar disorder type I 27.03% (range age: 25–49; male/female: 14/13), which are the psychiatric traits more frequently observed in the clinical centers used. The prevalence of lifetime substance use disorders (dual diagnosis) in the group of patients with schizophrenia was 40.28%, and in the patients with bipolar disorder was 57.72%. The mean age in suicide attempters was 35.55 years, the mean onset age of suicide attempt was 29.1 years (range 18–49 years), average schooling was 11.51 years (range 6–13 years); finally, 20 were males and 17 were females.

#### 2.3.1 Suicide attempt definition

We defined suicide attempt as a self‐harm behavior with at least some intent to end one's life. Suicide attempt was confirmed by trained psychiatrists using the Suicide Intent Scale in its Spanish version (Beck, Schuyler, & Herman, 1974; Fresan et al., 2015). When psychiatrists determined that the self‐injury behavior was performed without the intent to die, those individuals were excluded (Fresan et al., 2015; Gonzalez‐Castro et al., 2017).

### 2.4 Comparison group

The control group included random individuals who did not have active and/or passive suicide ideation/suicide attempt or family history of any suicide ideation/suicide attempt, this was determined during brief interviews performed by psychiatrists. Individuals were excluded if they had a concomitant medical or neurological illness and intellectual disability that would not allow them to consent. Of 200 individuals first selected, only 155 met all the selection criteria. The mean age in the control group was 34.37 years (range 24–55 years), the average schooling was 13.16 years (range 10–15 years); the sample comprised 76 males and 79 females.

### 2.5 Genotyping and imputation

Venous blood was taken from every individual and placed into EDTA containing tubes. DNA isolation was conducted in peripheral leukocytes following a previously published technique (Genis‐Mendoza et al., 2017; Lopez‐Narvaez et al., 2015; Molina‐Guzman et al., 2017). Direct genotyping was performed using the commercial platform Infinium PsychArray BeadChip (PsychChip, Illumina), following the protocol and conditions established by the provider. This platform includes around 560,000 variants distributed in the whole human genome and contains polymorphisms previously associated with psychiatric disorders. Intensity rates were converted to PLINK format files using the Genome Studio Platform. Subsequently, initial QC procedures on genotyping data were performed on PLINK (3). Individuals were excluded based on the following criteria: individuals and variants with a call rate lower than 95%; variants with minor allele frequency (MAF) lower than 5% and p‐value lower than 1e^−06^ for chi‐squared test for Hardy–Weinberg equilibrium (HWE). We also performed and identity‐by‐descent analysis, individuals with pihat ≥0.1 were removed from subsequent analyses. To have a higher coverage of polymorphisms in the genome, we performed an imputation process. Beagle with the multiethnic 1000 genomes project reference panel was used during the imputation process (Browning, Zhou, & Browning, 2018). After imputation, we used all variants with a MAF higher than 1% and we considered an imputed allelic dose higher than 0.4 as cut‐off. Imputation rendered a total of 4,835,917 single nucleotide polymorphisms.

### 2.6 Statistical analysis

#### 2.6.1 Generalized gene‐level genome‐wide association analysis

To evaluate the gene‐level association with suicide attempt phenotype, we performed a generalized gene‐level analysis recording any suicide attempt as a binary variable. The gene‐level genetic association analysis was performed in MAGMA (de Leeuw, Mooij, Heskes, & Posthuma, 2015). Data were analyzed under three different models: principal components regression, test of mean SNP association and test of top SNP associations. All models were adjusted by age, gender, main diagnosis (bipolar disease or schizophrenia) and the first five principal components of global ancestry. Global ancestry estimates were performed with a panel of ancestry informative markers previously published to differentiate American populations, the principal components were calculated using GCTA (Galanter et al., 2012; Yang, Lee, Goddard, & Visscher, 2011). For statistical significance, we considered ponderation of *p*‐values of the three different models of gene‐level analysis. A *p*‐value lower than 1e‐05 was considered as gene‐level significant and a *p*‐value lower than 1e‐04 was considered as gene‐level nominal significant.

#### 2.6.2 Variant‐level analysis

Once we found genes associated with suicide attempt through the gene‐level analysis, we performed a nested analysis of point variant on those genes (Wu & Zhi, 2013). For the variant‐level analysis, we extracted all variants imputed and performed genetic association testing suicide attempt by means of logistic regression analysis implemented in PLINK. All models were adjusted by age, gender, main diagnosis (bipolar disease or schizophrenia) and the first five principal components of global ancestry. Additive genetic model was applied to assess the significance of SNPs. A *p* < .05 was established as significance after Bonferroni correction.

#### 2.6.3 Prediction of functional analysis

We predicted the potential effect of gene polymorphisms to be associated with suicide attempt in psychiatric patients using bioinformatics tool RegulomeDB database (22955989).

#### 2.6.4 Enrichment analysis

Genes statistically associated with suicide attempt in our sample were included for Enrichment analysis using Enrichr (http://amp.pharm.mssm.edu/Enrichr/). Enrichr is an integrative web‐based and mobile software application that includes new gene‐set libraries, an alternative approach to rank enriched terms and various interactive visualization approaches to display enrichment results. Enrichr analyzed GO (gene ontology) biological process and pathways Kyoto Encyclopedia of Genes and Genomes – Genome (KEGG) in our sample, and detected genes/gene‐levels that were statistically associated with suicide attempt. For this, Enrichr uses three approaches: a) a standard Fisher exact test, b) a Fisher exact correction test based on intuition analysis to generate a *Z*‐score of the expected rank deviation and 3) a combination of both *p* values computed using the fisher exact test and the *Z*‐score; adjusted *p*‐values were obtained using the Benjamini‐Hochberg method for correction for multiple hypotheses testing.

## 3 RESULTS

### 3.1 Identification of novel genes associated with suicide attempt

In the genome‐wide gene‐level association analysis we found that Scavenger receptor class A member 5 (*SCARA5*) gene (OMIM accession number: 611306) was associated with suicide intent at gene‐level, with statistical significance (*p* = 6.77e‐06). Meanwhile, growth hormone secretagogue receptor (*GHSR*, *p* = 2.92e‐05, OMIM accession number: 601898), regulator of G protein signaling 10 (*RGS10*, *p* = 4.10e‐05, OMIM accession number: 602856) and serine/threonine kinase 33 (*STK33*, *p* = 8.23e‐05, OMIM accession number: 607670) were nominally associated with suicide attempt; Table 1. Also, see Figure S2.

**Table 1:** Summary of the gene‐level association to suicide attempt

Chromosome	Genomic position	Gene	# SNVs	Z‐stat	p‐value
3	172161081–172166246	GHSR	12	4.02	2.92e^−05^
8	27727399–27850369	SCARA5	352	4.35	6.77e^−06^
10	121259339–121302222	RGS10	128	3.93	4.10e^−05^
11	8413417–8615836	STK33	526	3.77	8.23e^−05^

Note

GHSR: NC_000003.12/ Gene ID 2693; SCARA5: NC_000008.11/ Gene ID 286133; RGS10: NC_000010.11/ Gene ID 6001; STK33: NC_000011.10/ Gene ID 65975.

### 3.2 Variant‐level genetic association

Once we found gene‐level associations, we performed a nested association test of all variants included in each gene. We found that 118 SNPs were associated with suicide attempt in this nested analysis. The variants associated with suicide attempt are reported in Table 2. Variants most highly associated with suicide attempt were rs565105 of *GSHR*, rs2685393 of *SCARA5*, rs561361616 of *RGS10* and rs11041981 of *STK33* (Tables 3 and 4).

**Table 2:** Variant genetic association with suicide attempt

Chromosome	Gene	SNP	BP	A1/A2	Clinical significance	Gene consequence	A1f cases	A1f controls	OR [CI 95%]	*p*‐value
3	*GHSR*	rs565105	172162117	G/T	Benign	3 prime UTR variant	0.44	0.18	3.35 [1.77–6.53]	2.33e^−04^
		rs482204	172162829	G/A	Benign	3 prime UTR variant	0.44	0.19	3.36 [1.75–6.46]	2.66e^−04^
		rs495225	172166033	G/A	Benign	Synonymous variant	0.44	0.20	3.04 [1.61–5.75]	6.40e^−04^
		rs2948694	172165163	G/A	Not reported	Intron variant	0.28	0.11	3.26 [1.54–6.91]	2.04e^−03^
8	*SCARA5*	rs2685393	27831887	C/T	Not reported	Intron variant	0.67	0.88	3.45 [1.82–6.57]	1.58e^−04^
10	*RGS10*	rs561361616	121294102	A/C	Not reported	Intron variant	0.74	0.47	0.20 [0.10–0.42]	2.08e^−05^
		rs1537577	121284536	C/T	Not reported	Intron variant	0.78	0.53	0.23 [0.11–0.47]	7.07e^−05^
		rs11198999	121285930	A/G	Not reported	Intron variant	0.76	0.51	0.25 [0.12–0.49]	7.84e^−05^
		rs9732576	121294035	G/A	Not reported	Intron variant	0.76	0.53	0.27 [0.14–0.54]	1.91e^−04^
		rs9732577	121294198	C/T	Not reported	Intron variant	0.76	0.53	0.27 [0.14–0.54]	1.91e^−04^
		rs2014999	121287641	C/A	Not reported	Intron variant	0.76	0.54	0.29 [0.15–0.57]	3.38e^−04^
		rs11199000	121288885	G/A	Not reported	Intron variant	0.76	0.54	0.29 [0.15–0.57]	3.38e^−04^
		rs17098973	121288501	G/A	Not reported	Intron variant	0.76	0.54	0.29 [0.15–0.57]	3.39e^−04^
11	*STK33*	rs11041981	8579208	T/C	Not reported	Intron variant	0.24	0.52	0.22 [0.11–0.44]	1.81e^−05^
		rs11041993	8596554	T/C	Not reported	Intron variant	0.25	0.53	0.22 [0.11–0.45]	2.27e^−05^
		rs11041994	8597866	C/A	Not reported	Intron variant	0.25	0.53	0.22 [0.11–0.45]	2.27e^−05^
		rs11041995	8598233	A/G	Not reported	Intron variant	0.25	0.53	0.22 [0.11–0.45]	2.27e^−05^
		rs11041997	8602016	G/A	Not reported	Intron variant	0.75	0.47	0.22 [0.11–0.45]	2.27e^−05^
		rs10840083	8608636	A/G	Not reported	Intron variant	0.25	0.53	0.22 [0.11–0.45]	2.27e^−05^
		rs10769918	8609710	C/T	Not reported	Intron variant	0.25	0.53	0.22 [0.11–0.45]	2.27e^−05^

Note

GHSR: NC_000003.12/ Gene ID 2693; SCARA5: NC_000008.11/ Gene ID 286133; RGS10: NC_000010.11/ Gene ID 6001; STK33: NC_000011.10/ Gene ID 65975

Abbreviations: A1, wild type allele; A1f, allele frequency of A1; A2, mutant allele; A2f, allele frequency of A2; BP, base pair.

**Table 3:** Biological process gene ontology implicated in genes associated with suicide attempt

Rank	Biological Process (accession number)	*p*‐value	Adjusted *p*‐value	*Z*‐score	Combined score
1	growth hormone secretion (GO:0030252)	0.001399	0.01419	−3.2	21.05
2	positive regulation of multicellular organism growth (GO:0040018)	0.001599	0.01419	−2.78	17.91
3	negative regulation of interleukin−1 production (GO:0032692)	0.001799	0.01419	−2.76	17.45
4	iron ion transport (GO:0006826)	0.002598	0.01419	−2.92	17.4
5	regulation of tumor necrosis factor biosynthetic process (GO:0042534)	0.002398	0.01419	−2.71	16.34
6	negative regulation of interleukin−1 beta production (GO:0032691)	0.002997	0.01419	−2.48	14.39
7	regulation of interleukin−6 biosynthetic process (GO:0045408)	0.001999	0.01419	−2.23	13.87
8	regulation of interleukin−1 beta production (GO:0032651)	0.002797	0.01419	−2.3	13.54
9	G protein‐coupled acetylcholine receptor signaling pathway (GO:0007213)	0.003396	0.01419	−2.35	13.38
10	acetylcholine receptor signaling pathway (GO:0095500)	0.003994	0.01419	−2.23	12.31
11	regulation of multicellular organism growth (GO:0040014)	0.003595	0.01419	−2.11	11.88
12	cellular iron ion homeostasis (GO:0006879)	0.01135	0.02327	−2.62	11.73
13	negative regulation of tumor necrosis factor production (GO:0032720)	0.007181	0.01681	−2.32	11.45
14	protein homotrimerization (GO:0070207)	0.004592	0.01419	−2.11	11.37
15	peptide hormone secretion (GO:0030072)	0.00519	0.01419	−2.07	10.9
16	negative regulation of cytokine biosynthetic process (GO:0042036)	0.004393	0.01419	−1.95	10.6
17	actin polymerization or depolymerization (GO:0008154)	0.00738	0.01681	−1.93	9.5
18	positive regulation of developmental growth (GO:0048639)	0.01036	0.02236	−2.07	9.44
19	protein trimerization (GO:0070206)	0.006584	0.01681	−1.72	8.62
20	negative regulation of interleukin−6 production (GO:0032715)	0.004991	0.01419	−1.52	8.07
21	negative regulation of inflammatory response (GO:0050728)	0.01571	0.02751	−1.83	7.59
22	negative regulation of defense response (GO:0031348)	0.0161	0.02751	−1.74	7.17
23	peptidyl‐threonine modification (GO:0018210)	0.01788	0.02932	−1.73	6.98
24	iron ion homeostasis (GO:0055072)	0.01274	0.02487	−1.56	6.8
25	cellular transition metal ion homeostasis (GO:0046916)	0.01867	0.02944	−1.57	6.24
26	protein autophosphorylation (GO:0046777)	0.03474	0.04189	−1.79	6.03
27	positive regulation of hydrolase activity (GO:0051345)	0.03416	0.04189	−1.7	5.74
28	positive regulation of multicellular organismal process (GO:0051240)	0.03999	0.04315	−1.69	5.44
29	peptidyl‐threonine phosphorylation (GO:0018107)	0.01373	0.02559	−1.23	5.27
30	peptidyl‐serine phosphorylation (GO:0018105)	0.02888	0.03947	−1.44	5.1
31	endocytosis (GO:0006897)	0.05177	0.05442	−1.65	4.89
32	peptidyl‐serine modification (GO:0018209)	0.03377	0.04189	−1.44	4.89
33	protein homooligomerization (GO:0051260)	0.03766	0.04216	−1.46	4.78
34	negative regulation of response to external stimulus (GO:0032102)	0.02143	0.03254	−1.19	4.58
35	inorganic cation transmembrane transport (GO:0098662)	0.02791	0.03945	−1.26	4.51
36	actin filament organization (GO:0007015)	0.02398	0.03512	−1.16	4.32
37	vesicle‐mediated transport (GO:0016192)	0.07971	0.0817	−1.61	4.08
38	regulation of GTPase activity (GO:0043087)	0.03727	0.04216	−1.21	3.97
39	positive regulation of GTPase activity (GO:0043547)	0.03805	0.04216	−1.17	3.84
40	regulation of inflammatory response (GO:0050727)	0.03299	0.04189	−1.01	3.44
41	protein phosphorylation (GO:0006468)	0.09093	0.09093	−1.07	2.56

**Table 4:** Pathways implicated in genes associated with suicide attempt

Rank	KEGG pathway (accession number)	*p*‐value	Adjusted *p*‐value	*Z*‐score	Combined score
1	cAMP signaling pathway (hsa04024)	0.03921	0.05426	−1.94	6.3
2	Neuroactive ligand–receptor interaction (hsa04080)	0.05426	0.05426	−1.87	5.45

### 3.3 Prediction of functional analysis of variants associated with suicide attempt

For the 118 variants associated with suicide attempt, we determined the type of each variant. We observed that 10 variants were short insertion/deletion and 108 were single nucleotide variants. Regarding gene location of these variants, we found 113 to be intronic, two were located in untranslated regions (rs565105 and rs482204), two were near the region of splicing (rs7934396 and rs2289921) and one was a synonymous variant (rs495225). As variants were located on noncoding regions with no effects on protein structure, we searched for variants in the RegulomeDB database and only reported those with the highest scores (scores only in 1). The following variants had the highest scores and all laid in gene *STK33*: rs1374262 (score: 1f, TF: *FOXA1*, eQTL: *EIF3F*), rs7101471 (score: 1d, TF: *SPI1*, eQTL: *EIF3F*), rs7939731 (score: 1d, TF: *ESR1*, eQTL: *EIF3F*), rs10769915 (score: 1f, eQTL: c*11orf17*), rs10160430 (score: 1f, TF: *SETDB1*, eQTL: *c11orf17*).

### 3.4 Enrichment analysis

We explored gene ontology and pathways involved in genes statistically associated with suicide attempt in our population. Derived from this, we obtained 41 gene ontologies of biological processes; after adjusting *p* values, 38 gene ontologies of biological processes showed statistical significance (*p* < .05). Regarding pathways, we only identified two: cAMP signaling pathway and neuroactive ligand–receptor interaction, none of them maintained statistical significance after adjusting *p* values.

## 4 DISCUSSION

Nowadays, it is well known that there are a variety of biological risk factors for suicide attempt. GWAS allow to explore biological systems that have not been studied yet. Therefore, our aim was to conduct a gene‐level association study in Mexican individuals diagnosed with schizophrenia or bipolar disorders who had attempted suicide, in order to find genetic determinants that could increase the risk of SA behavior.

Initially, our outcomes revealed novel genes associated with SA in Mexican individuals with psychiatric disorders; for instance, *SCARA5* was significantly associated. In 2006, *SCARA5* was identified as the novel member of the SR family by Jing et al, and its gene is located on chromosome 8p21.1 (Jiang, Oliver, Davies, & Platt, 2006). The association with SA could be through scavenger receptors class A (SR‐A) that initiates inflammatory responses; *SCARA5* expression itself is associated with the activation/polarization of microglia (Tang et al., 2018). Activated microglia produce cytokines and chemokines that impact synaptic plasticity, neurotransmitter metabolism, and neurocircuits relevant to mood regulation (Isgren et al., 2017). Moreover, increased transcriptome variability of SCARA5 has been observed in the temporal cortex of patents with autism (Garbett et al., 2008); one can speculate that this pathway participates in the role of SR‐A in microglia inflammatory response, which is observed in some neuropsychiatric diseases (Brites & Fernandes, 2015; Cornejo et al., 2018). The association of *SCARA5* in psychiatric patients with suicide attempt had not been reported before; nevertheless, previous evidence of association with psychiatric disorders has been found in GWAS and schizophrenia and depression, among others (Carboni et al., 2018; Rincon‐Cortes et al., 2015; Xu et al., 2013). Therefore, we could infer that *SCARA5* has a small participation in SA behavior. Future validation of these results could provide a better understanding of risk factors that influence the development of suicide behavior and suicide attempt.

This preliminary study also revealed that *GHSR* is associated with SA in our Mexican population with psychiatric disorders. *GHSR* encodes a member of the G protein‐coupled receptor family, which is located on chromosome region 3q26.31 (Cabral, Lopez Soto, Epelbaum, & Perello, 2017; Valentina, Susana, & Jesica, 2018). It has been reported that GHSR can heterodimerize with other G protein‐coupled receptors, such as serotonin receptor 2C and dopamine receptors D1 and D2, which are strongly associated with SB (Cabral et al., 2017; Genis‐Mendoza et al., 2017). A relation between GHSR and SA behavior could be due to *GHSR* expression mainly in the pituitary gland and other specific areas of the central nervous system; for instance, it is highly distributed in hypothalamus, which is an important region that controls neuroendocrine functions (Kim et al., 2003; Valentina et al., 2018). High *GHSR* expression is also found in hippocampus, amygdala, and ventral tegmental area, which have an important participation in mood regulation (Huang et al., 2017). Derived from this, we could suggest that GHSR activity impacts strongly on neuronal activity, which entails a possible physiological and behavioral effect. These outcomes would help the search for candidate gene regions associated with predisposition to SA behavior.

We also found association between other genes and suicide attempt, such as *RGS‐10* located on chromosome region 10q26.11. The regulator of G protein signaling (RGS) plays a crucial role in modulating GPCR signaling through its GTPase‐activating protein activity toward Gα subunits (Lee & Tansey, 2015). Due to various effects of GPCR signaling downstream, RGS proteins have been implicated in a number of diseases, ranging from cancer to CNS disorders (Hishimoto et al., 2004; Lee & Tansey, 2015; Stuart Gibbons et al., 2008). In this sense, it has been suggested that the dysfunction of neuronal signal transduction via G protein is involved in the pathophysiology of schizophrenia and may also be in bipolar disorder (Stuart Gibbons et al., 2008). Some scholars propose that some polymorphisms of *RGS10* which cause amino acid substitutions play a role in the biological susceptibility to these diseases through structural changes at protein level altering protein function; for instance, altering GAP activity. (Lee & Tansey, 2015). However, this must be taken with caution as Rivero et al. (2013) evaluated the expression of RGS4 and RGS10 proteins in postmortem brains of individuals with psychiatric disorders, and found unaltered membranes of RGS4 and cytosolic RGS10 proteins levels in schizophrenia and major depression in a mixed population from Switzerland and Spain (Rivero et al., 2013). Further studies in Mexican population are needed in order to elucidate RGS10 functional status.

The last gene we found to be associated with SB in our analysis was *STK33*. STK33 was first discovered and classified as a serine/threonine‐protein kinase putatively related to the Ca2+/calmodulin‐dependent kinase family (CAMK) in 2001 (Mujica, Hankeln, & Schmidt, 2001). *STK33* is located on chromosome region 11p15.3; its entire functions have not been elucidated yet; however, there are some suggestions (Brauksiepe, Baumgarten, Reuss, & Schmidt, 2014). For example, Reuss, Brauksiepe, Disque‐Kaiser, & Olivier, 2017 evaluated the expression and presence of STK33 protein in neuronal structures of central nervous system in rats and hamsters and observed large STK33‐immunoreactive axonal projections from magnocellular hypothalamus to the neurohypophysis (Reuss et al., 2017). Hence, this functional connection suggests a bridge from neuronal to humoral regulation of the endocrine system. Subsequently, our results are important due to a possible implication of STK33 in SA; this gene deserves further attention in association studies of SB in Mexican population. With regards of predicting the type of variants that were associated with SA, they were mainly intronic, located on untranslated regions or near splicing regions, only one was a synonymous variant change. Therefore, we could say that even though there were no variants located on coding regions, these variants may still alter gene expression.

We want to emphasize that our findings have some limitations. Firstly, the sample size could be considered small and therefore has a low statistical power, decreasing the likelihood to detect small effects of candidate genes. The previous limitation is linked with the fact that we could not search for specific endophenotypes that may be involved in the pathogenesis of suicide attempt in our population. Future studies will need to address this issue either through incorporating covariates that help to improve the power of analysis (e.g. psychosocial risk factor for SA behaviors), stratification of characteristic phenotypes of suicide attempters (e.g. early onset for SA behavior) or increasing the sample size. We want to emphasize that we used the DSM IV criteria instead of the newest version recently published (DSM‐V); therefore, we did not take into account neurodegenerative conditions and substance use, which could be considered as a limitation, as some of the individuals included had a substance use disorder. This co‐occurring psychiatric disorders with substance use had been observed in other Mexican samples (Marin‐Navarrete et al., 2016); in fact, in Mexico it is common that individuals with psychiatric disorders (schizophrenia, bipolar among others) consume alcohol or other drugs (Fresan et al., 2015; Gonzalez‐Castro et al., 2016). Then, we recommend taking into consideration this factor when interpreting the study outcomes. Another limitation is that we did not evaluate the impact that rare variants or copy number variants could have in our population. Nevertheless, our study suggests some candidate genes that could be used as tools to identify patients with serious risk of SA in future studies. Finally, we did not analyze environmental factors that may interact with genetic factors and increase the risk of SB (e.g. parental loss).

To sum up, our outcomes at gene‐level revealed that *SCARA5*, *GHSR*, *RGS10*, and *STK33* are candidate genes that could participate as risk biomarkers for suicide attempt in our Mexican psychiatric sample. Our findings should be judged as preliminary findings due to the limitations above mentioned. Nonetheless, we consider that this study could lead to the making of a valid hypothesis of genetic influence to develop suicide attempt in Mexican individuals with psychiatric disorders in further larger‐scale analyses.

## CONFLICT OF INTEREST

None declared.

## AUTHOR CONTRIBUTIONS

Conceptualization: Alma Delia Genis‐Mendoza and Humberto Nicolini. Data curation: Emmanuel Sarmiento. Funding acquisition: Humberto Nicolini. Investigation: Thelma Beatriz González‐Castro. Methodology, José Jaime Martínez‐Magaña. Project administration: Humberto Nicolini. Resources: Isela Esther Juárez‐Rojop. Software: José Jaime Martínez‐Magaña. Supervision: Isela Esther Juárez‐Rojop. Validation: Thelma Beatriz González‐Castro, Alma Delia Genis‐Mendoza and Humberto Nicolini. Visualization, Emmanuel Sarmiento. Writing – original draft: Carlos Alfonso Tovilla‐Zárate. Writing – review and editing: Thelma Beatriz González‐Castro.

## Data Availability

None applicable.

## References

[mgg3983-bib-0001] Beck, A. T. , Schuyler, D. , & Herman, I. (1974). Development of suicidal intent scales. Oxford, UK: Charles Press Publishers.

[mgg3983-bib-0002] Borges, G. , Orozco, R. , Villatoro, J. , Medina‐Mora, M. E. , Fleiz, C. , & Diaz‐Salazar, J. (2019). Suicide ideation and behavior in Mexico: Encodat 2016. Salud Publica de Mexico, 61(1), 6–15. 10.21149/9351 30753768

[mgg3983-bib-0003] Brauksiepe, B. , Baumgarten, L. , Reuss, S. , & Schmidt, E. R. (2014). Co‐localization of serine/threonine kinase 33 (Stk33) and vimentin in the hypothalamus. Cell and Tissue Research, 355(1), 189–199. 10.1007/s00441-013-1721-8 24057876

[mgg3983-bib-0004] Brites, D. , & Fernandes, A. (2015). Neuroinflammation and depression: Microglia activation, extracellular microvesicles and microRNA dysregulation. Front Cell Neurosci, 9, 476 10.3389/fncel.2015.00476 26733805 PMC4681811

[mgg3983-bib-0005] Browning, B. L. , Zhou, Y. , & Browning, S. R. (2018). A one‐penny imputed genome from next‐generation reference panels. American Journal of Human Genetics, 103(3), 338–348. 10.1016/j.ajhg.2018.07.015 30100085 PMC6128308

[mgg3983-bib-0006] Cabral, A. , Lopez Soto, E. J. , Epelbaum, J. , & Perello, M. (2017). Is Ghrelin synthesized in the central nervous system? International Journal of Molecular Sciences, 18(3), 10.3390/ijms18030638 PMC5372651 28294994

[mgg3983-bib-0007] Carboni, L. , Marchetti, L. , Lauria, M. , Gass, P. , Vollmayr, B. , Redfern, A. , … Mathé, A. A. (2018). Cross‐species evidence from human and rat brain transcriptome for growth factor signaling pathway dysregulation in major depression. Neuropsychopharmacology, 43(10), 2134–2145. 10.1038/s41386-018-0117-6 29950584 PMC6098161

[mgg3983-bib-0008] Cornejo, F. , Vruwink, M. , Metz, C. , Muñoz, P. , Salgado, N. , Poblete, J. , … von Bernhardi, R. (2018). Scavenger Receptor‐A deficiency impairs immune response of microglia and astrocytes potentiating Alzheimer's disease pathophysiology. Brain, Behavior, and Immunity, 69, 336–350. 10.1016/j.bbi.2017.12.007 29246456

[mgg3983-bib-0009] de Leeuw, C. A. , Mooij, J. M. , Heskes, T. , & Posthuma, D. (2015). MAGMA: Generalized gene‐set analysis of GWAS data. PLoS Computational Biology, 11(4), e1004219 10.1371/journal.pcbi.1004219 25885710 PMC4401657

[mgg3983-bib-0010] Eftekharian, M. M. , Noroozi, R. , Omrani, M. D. , Sharifi, Z. , Komaki, A. , Taheri, M. , & Ghafouri‐Fard, S. (2018). Single‐Nucleotide polymorphisms in interleukin 6 (IL‐6) gene are associated with suicide behavior in an iranian population. Journal of Molecular Neuroscience, 66(3), 414–419. 10.1007/s12031-018-1190-3 30269203

[mgg3983-bib-0011] Fresán, A. , González‐Castro, T. B. , Peralta‐Jiménez, Y. , Juárez‐Rojop, I. , Pool‐García, S. , Velázquez‐Sánchez, M. P. , … Tovilla‐Zárate, C. A. (2015). Gender differences in socio‐demographic, clinical characteristics and psychiatric diagnosis in/of suicide attempters in a Mexican population. Acta Neuropsychiatr, 27(3), 182–188. 10.1017/neu.2015.6 25686910

[mgg3983-bib-0012] Fu, Q. , Heath, A. C. , Bucholz, K. K. , Nelson, E. C. , Glowinski, A. L. , Goldberg, J. , … Eisen, S. A. (2002). A twin study of genetic and environmental influences on suicidality in men. Psychological Medicine, 32(1), 11–24. 10.1017/S0033291701004846 11883722

[mgg3983-bib-0013] Galanter, J. M. , Fernandez‐Lopez, J. C. , Gignoux, C. R. , Barnholtz‐Sloan, J. , Fernandez‐Rozadilla, C. , Via, M. , … Carracedo, A. (2012). Development of a panel of genome‐wide ancestry informative markers to study admixture throughout the Americas. PLoS Genetics, 8(3), e1002554 10.1371/journal.pgen.1002554 22412386 PMC3297575

[mgg3983-bib-0014] Galfalvy, H. , Haghighi, F. , Hodgkinson, C. , Goldman, D. , Oquendo, M. A. , Burke, A. , … Mann, J. J. (2015). A genome‐wide association study of suicidal behavior. American Journal of Medical Genetics. Part B, Neuropsychiatric Genetics, 168(7), 557–563. 10.1002/ajmg.b.32330 26079190

[mgg3983-bib-0015] Galfalvy, H. , Zalsman, G. , Huang, Y.‐Y. , Murphy, L. , Rosoklija, G. , Dwork, A. J. , … Mann, J. J. (2013). A pilot genome wide association and gene expression array study of suicide with and without major depression. The World Journal of Biological Psychiatry, 14(8), 574–582. 10.3109/15622975.2011.597875 22059935 PMC3493880

[mgg3983-bib-0016] Garbett, K. , Ebert, P. J. , Mitchell, A. , Lintas, C. , Manzi, B. , Mirnics, K. , & Persico, A. M. (2008). Immune transcriptome alterations in the temporal cortex of subjects with autism. Neurobiology of Diseases, 30(3), 303–311. 10.1016/j.nbd.2008.01.012 PMC2693090 18378158

[mgg3983-bib-0017] Genis‐Mendoza, A. D. , López‐Narvaez, M. L. , Tovilla‐Zárate, C. A. , Sarmiento, E. , Chavez, A. , Martinez‐Magaña, J. J. , … Nicolini, H. (2017). Association between polymorphisms of the DRD2 and ANKK1 genes and suicide attempt: A preliminary case‐control study in a Mexican population. Neuropsychobiology, 76(4), 193–198. 10.1159/000490071 29966133

[mgg3983-bib-0018] Glowinski, A. L. , Bucholz, K. K. , Nelson, E. C. , Fu, Q. , Madden, P. A. , Reich, W. , & Heath, A. C. (2001). Suicide attempts in an adolescent female twin sample. Journal of the American Academy of Child and Adolescent Psychiatry, 40(11), 1300–1307. 10.1097/00004583-200111000-00010 11699804 PMC1474069

[mgg3983-bib-0019] González‐Castro, T. B. , Hernández‐Díaz, Y. , Tovilla‐Zárate, C. A. , González‐Gutiérrez, K. P. , Fresán, A. , Juárez‐Rojop, I. E. , … Genis, A. (2016). Differences by gender in completed suicides in a Mexican population: A psychological autopsy study. Journal of Forensic and Legal Medicine, 38, 70–74. 10.1016/j.jflm.2015.11.019 26717248

[mgg3983-bib-0020] González‐Castro, T. B. , Tovilla‐Zárate, C. A. , Hernández‐Díaz, Y. , Juárez‐Rojop, I. E. , León‐Garibay, A. G. , Guzmán‐Priego, C. G. , … Frésan, A. (2017). Characteristics of Mexican children and adolescents who died by suicide: A study of psychological autopsies. Journal of Forensic and Legal Medicine, 52, 236–240. 10.1016/j.jflm.2017.10.002 29035840

[mgg3983-bib-0021] Hishimoto, A. , Shirakawa, O. , Nishiguchi, N. , Aoyama, S. , Ono, H. , Hashimoto, T. , & Maeda, K. (2004). Novel missense polymorphism in the regulator of G‐protein signaling 10 gene: Analysis of association with schizophrenia. Psychiatry and Clinical Neurosciences, 58(5), 579–581. 10.1111/j.1440-1819.2004.01303.x 15482592

[mgg3983-bib-0022] Huang, H.‐J. , Zhu, X.‐C. , Han, Q.‐Q. , Wang, Y.‐L. , Yue, N. A. , Wang, J. , … Yu, J. (2017). Ghrelin alleviates anxiety‐ and depression‐like behaviors induced by chronic unpredictable mild stress in rodents. Behavioral Brain Research, 326, 33–43. 10.1016/j.bbr.2017.02.040 28245976

[mgg3983-bib-0023] Isgren, A. , Sellgren, C. , Ekman, C.‐J. , Holmén‐Larsson, J. , Blennow, K. , Zetterberg, H. , … Landén, M. (2017). Markers of neuroinflammation and neuronal injury in bipolar disorder: Relation to prospective clinical outcomes. Brain, Behavior, and Immunity, 65, 195–201. 10.1016/j.bbi.2017.05.002 28483660

[mgg3983-bib-0024] Jiang, Y. , Oliver, P. , Davies, K. E. , & Platt, N. (2006). Identification and characterization of murine SCARA5, a novel class A scavenger receptor that is expressed by populations of epithelial cells. Journal of Biological Chemistry, 281(17), 11834–11845. 10.1074/jbc.M507599200 16407294

[mgg3983-bib-0025] Kim, K. , Sanno, N. , Arai, K. , Takano, K. , Yasufuku‐Takano, J. , … Shibasaki, T. (2003). Ghrelin mRNA and GH secretagogue receptor mRNA in human GH‐producing pituitary adenomas is affected by mutations in the alpha subunit of G protein. Clinical Endocrinology ‐ Oxford, 59(5), 630–636. 10.1046/j.1365-2265.2003.01898.x 14616888

[mgg3983-bib-0026] Kimbrel, N. A. , Garrett, M. E. , Dennis, M. F. , Hauser, M. A. , Ashley‐Koch, A. E. , & Beckham, J. C. (2018). A genome‐wide association study of suicide attempts and suicidal ideation in U.S. military veterans. Psychiatry Research, 269, 64–69. 10.1016/j.psychres.2018.07.017 30145303 PMC6207450

[mgg3983-bib-0027] Lasky‐Su, J. , & Lange, C. (2010). Statistical challenges for genome‐wide association studies of suicidality using family data. European Psychiatry, 25(5), 307–309. 10.1016/j.eurpsy.2009.12.019 20447807 PMC2925169

[mgg3983-bib-0028] Lee, J. K. , & Tansey, M. G. (2015). Physiology of RGS10 in neurons and immune cells. Progress in Molecular Biology and Translational Science, 133, 153–167. 10.1016/bs.pmbts.2015.01.005 26123306

[mgg3983-bib-0029] López‐Narváez, M. L. , Tovilla‐Zárate, C. A. , González‐Castro, T. B. , Juárez‐Rojop, I. , Pool‐García, S. , Genis, A. , … Fresán, A. (2015). Association analysis of TPH‐1 and TPH‐2 genes with suicidal behavior in patients with attempted suicide in Mexican population. Comprehensive Psychiatry, 61, 72–77. 10.1016/j.comppsych.2015.05.002 26028568

[mgg3983-bib-0030] Marin‐Navarrete, R. , Medina‐Mora, M. E. , Horigian, V. E. , Salloum, I. M. , Villalobos‐Gallegos, L. , & Fernandez‐Mondragon, J. (2016). Co‐occurring disorders: A challenge for Mexican community‐based residential care facilities for substance use. Journal of Dual Diagnosis, 12(3–4), 261–270. 10.1080/15504263.2016.1220207 27494051 PMC6929207

[mgg3983-bib-0031] Melroy‐Greif, W. E. , Gizer, I. R. , Wilhelmsen, K. C. , & Ehlers, C. L. (2017). Genetic influences on evening preference overlap with those for bipolar disorder in a sample of Mexican Americans and American Indians. Twin Research and Human Genetics, 20(6), 499–510. 10.1017/thg.2017.62 29192581 PMC6013261

[mgg3983-bib-0032] Mirkovic, B. , Laurent, C. , Podlipski, M. A. , Frebourg, T. , Cohen, D. , & Gerardin, P. (2016). Genetic association studies of suicidal behavior: A review of the past 10 years, progress, limitations, and future directions. Frontiers in Psychiatry, 7, 158 10.3389/fpsyt.2016.00158 27721799 PMC5034008

[mgg3983-bib-0033] Molina‐Guzman, G. , Gonzalez‐Castro, T. B. , Hernandez Diaz, Y. , Tovilla‐Zarate, C. A. , Juarez‐Rojop, I. E. , Guzman‐Priego, C. G. , … Rodriguez‐Perez, J. M. (2017). Gender differences in the association between HTR2C gene variants and suicidal behavior in a Mexican population: A case‐control study. Neuropsychiatric Disease and Treatment, 13, 559–566. 10.2147/ndt.s122024 28260903 PMC5328611

[mgg3983-bib-0034] Mujica, A. O. , Hankeln, T. , & Schmidt, E. R. (2001). A novel serine/threonine kinase gene, STK33, on human chromosome 11p15.3. Gene, 280(1–2), 175–181. 11738831 10.1016/s0378-1119(01)00780-6

[mgg3983-bib-0035] Mullins, N. , Perroud, N. , Uher, R. , Butler, A. W. , Cohen‐Woods, S. , Rivera, M. , … Lewis, C. M. (2014). Genetic relationships between suicide attempts, suicidal ideation and major psychiatric disorders: A genome‐wide association and polygenic scoring study. American Journal of Medical Genetics. Part B, Neuropsychiatric Genetics, 165B(5), 428–437. 10.1002/ajmg.b.32247 PMC4309466 24964207

[mgg3983-bib-0036] Organiztion, W. H. (2019). Global Health Observatory data. https://www.who.int/gho/mental_health/suicide_rates/en/ Accesed 15 August, 2019.

[mgg3983-bib-0037] Perlis, R. H. , Huang, J. , Purcell, S. , Fava, M. , Rush, A. J. , Sullivan, P. F. , … Smoller, J. W. (2010). Genome‐wide association study of suicide attempts in mood disorder patients. American Journal of Psychiatry, 167(12), 1499–1507. 10.1176/appi.ajp.2010.10040541 21041247 PMC3795390

[mgg3983-bib-0038] Powers, A. , Almli, L. , Smith, A. , Lori, A. , Leveille, J. , Ressler, K. J. , … Bradley, B. (2016). A genome‐wide association study of emotion dysregulation: Evidence for interleukin 2 receptor alpha. Journal of Psychiatric Research, 83, 195–202. 10.1016/j.jpsychires.2016.09.006 27643478 PMC5896292

[mgg3983-bib-0039] Reuss, S. , Brauksiepe, B. , Disque‐Kaiser, U. , & Olivier, T. (2017). Serine/threonine‐kinase 33 (Stk33) ‐ Component of the neuroendocrine network? Brain Research, 1655, 152–160. 10.1016/j.brainres.2016.11.006 27840186

[mgg3983-bib-0040] Rincon‐Cortes, M. , Barr, G. A. , Mouly, A. M. , Shionoya, K. , Nunez, B. S. , & Sullivan, R. M. (2015). Enduring good memories of infant trauma: Rescue of adult neurobehavioral deficits via amygdala serotonin and corticosterone interaction. Proceedings of the National Academy of Sciences USA, 112(3), 881–886. 10.1073/pnas.1416065112 PMC4311810 25561533

[mgg3983-bib-0041] Rivero, G. , Gabilondo, A. M. , Garcia‐Sevilla, J. A. , Callado, L. F. , La Harpe, R. , Morentin, B. , & Meana, J. J. (2013). Brain RGS4 and RGS10 protein expression in schizophrenia and depression. Effect of Drug Treatment. Psychopharmacology (Berl), 226(1), 177–188. 10.1007/s00213-012-2888-5 23093381

[mgg3983-bib-0042] Schneider, F. , & Grozinger, M. (2010). Psychiatric illness through the lifespan. European Archives of Psychiatry and Clinical Neuroscience, 260(Suppl 2), S79–S80. 10.1007/s00406-010-0163-5 20960001

[mgg3983-bib-0043] Sokolowski, M. , Wasserman, J. , & Wasserman, D. (2014). Genome‐wide association studies of suicidal behaviors: A review. European Neuropsychopharmacology, 24(10), 1567–1577. 10.1016/j.euroneuro.2014.08.006 25219938

[mgg3983-bib-0044] Sokolowski, M. , Wasserman, J. , & Wasserman, D. (2016a). Polygenic associations of neurodevelopmental genes in suicide attempt. Molecular Psychiatry, 21(10), 1381–1390. 10.1038/mp.2015.187 26666204

[mgg3983-bib-0045] Sokolowski, M. , Wasserman, J. , & Wasserman, D. (2016b). Rare CNVs in suicide attempt include schizophrenia‐associated loci and neurodevelopmental genes: A pilot genome‐wide and family‐based study. PLoS ONE, 11(12), e0168531 10.1371/journal.pone.0168531 28030616 PMC5193342

[mgg3983-bib-0046] Stein, M. B. , Ware, E. B. , Mitchell, C. , Chen, C.‐Y. , Borja, S. , Cai, T. , … Smoller, J. W. (2017). Genomewide association studies of suicide attempts in US soldiers. American Journal of Medical Genetics. Part B, Neuropsychiatric Genetics, 174(8), 786–797. 10.1002/ajmg.b.32594 PMC5685938 28902444

[mgg3983-bib-0047] Stuart Gibbons, A. , Scarr, E. , McOmish, C. E. , Hannan, A. J. , Thomas, E. A. , & Dean, B. (2008). Regulator of G‐protein signalling 4 expression is not altered in the prefrontal cortex in schizophrenia. Australian and New Zealand Journal of Psychiatry, 42(8), 740–745. 10.1080/00048670802206338 18622782

[mgg3983-bib-0048] Tang, T. , Valenzuela, A. , Petit, F. , Chow, S. , Leung, K. , Gorin, F. , … Dhenain, M. (2018). In vivo MRI of functionalized iron oxide nanoparticles for brain inflammation. Contrast Media & Molecular Imaging, 2018, 3476476 10.1155/2018/3476476 30079001 PMC6036843

[mgg3983-bib-0049] Valentina, M. D. , Susana, R. S. , & Jesica, R. (2018). GHSR constitutive activity impairs voltage‐gated calcium channel (CaV )‐dependent inhibitory neurotransmission in hippocampal neurons. Journal of Physiology, 596(2), 5415–5428. 10.1113/jp276256 30199095 PMC6235945

[mgg3983-bib-0050] Wang, L. , Jia, P. , Wolfinger, R. D. , Chen, X. , & Zhao, Z. (2011). Gene set analysis of genome‐wide association studies: Methodological issues and perspectives. Genomics, 98(1), 1–8. 10.1016/j.ygeno.2011.04.006 21565265 PMC3852939

[mgg3983-bib-0051] Willour, V. L. , Seifuddin, F. , Mahon, P. B. , Jancic, D. , Pirooznia, M. , Steele, J. , … Potash, J. B. (2012). A genome‐wide association study of attempted suicide. Molecular Psychiatry, 17(4), 433–444. 10.1038/mp.2011.4 21423239 PMC4021719

[mgg3983-bib-0052] Wu, G. , & Zhi, D. (2013). Pathway‐based approaches for sequencing‐based genome‐wide association studies. Genetic Epidemiology, 37(5), 478–494. 10.1002/gepi.21728 23650134 PMC3856324

[mgg3983-bib-0053] Xu, C. , Aragam, N. , Li, X. , Villla, E. C. , Wang, L. , Briones, D. , … Wang, K. S. (2013). BCL9 and C9orf5 are associated with negative symptoms in schizophrenia: Meta‐analysis of two genome‐wide association studies. PLoS ONE, 8(1), e51674 10.1371/journal.pone.0051674 23382809 PMC3558516

[mgg3983-bib-0054] Yang, J. , Lee, S. H. , Goddard, M. E. , & Visscher, P. M. (2011). GCTA: A tool for genome‐wide complex trait analysis. American Journal of Human Genetics, 88(1), 76–82. 10.1016/j.ajhg.2010.11.011 21167468 PMC3014363

[mgg3983-bib-0055] Zai, C. C. , Gonçalves, V. F. , Tiwari, A. K. , Gagliano, S. A. , Hosang, G. , de Luca, V. , … Kennedy, J. L. (2015). A genome‐wide association study of suicide severity scores in bipolar disorder. Journal of Psychiatric Research, 65, 23–29. 10.1016/j.jpsychires.2014.11.002 25917933

