# Supplementary material for: Gene‐level genome‐wide association analysis of suicide attempt, a preliminary study in a psychiatric Mexican population
Source: Mol Genet Genomic Med. 2019 Oct 2;7(12):e983. doi: 10.1002/mgg3.983 (PMC6900393; doi:10.1002/mgg3.983)

Figure 1. Flowchart of the selection criteria of suicide attempters.

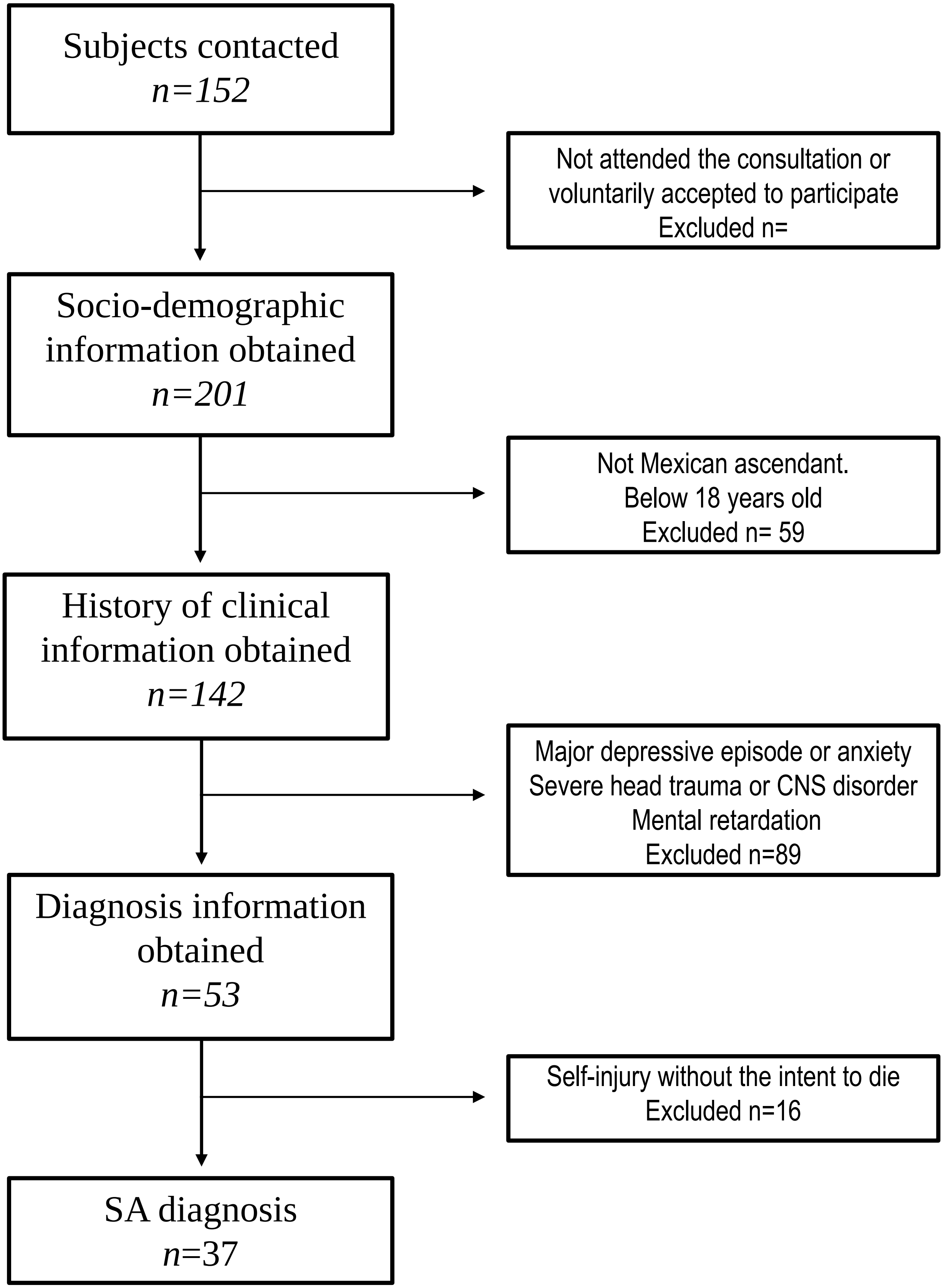

Figure 2. Manhattan plot of P values in preliminary GWAS of suicide attempters in Mexican psychiatric patients vs. controls

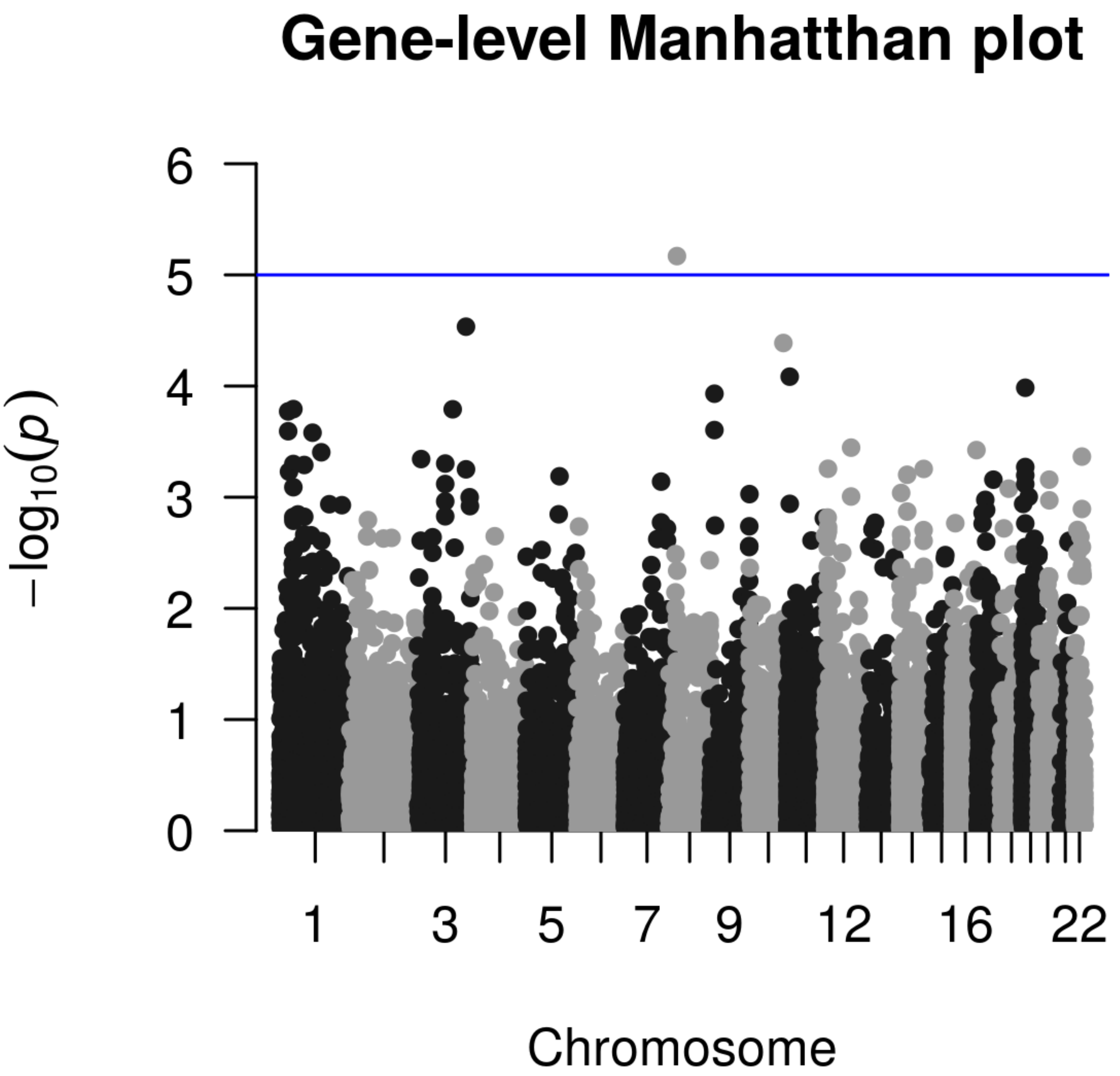

Supplement: Supplementary file 1 [file MGG3-7-e983-s001.pdf]
